# Reinspection of a Clinical Proteomics Tumor Analysis Consortium (CPTAC) Dataset with Cloud Computing Reveals Abundant Post-Translational Modifications and Protein Sequence Variants

**DOI:** 10.3390/cancers13205034

**Published:** 2021-10-09

**Authors:** Amol Prakash, Lorne Taylor, Manu Varkey, Nate Hoxie, Yassene Mohammed, Young Ah Goo, Scott Peterman, Abhay Moghekar, Yuting Yuan, Trevor Glaros, Joel R. Steele, Pouya Faridi, Shashwati Parihari, Sanjeeva Srivastava, Joseph J. Otto, Julius O. Nyalwidhe, O. John Semmes, Michael F. Moran, Anil Madugundu, Dong Gi Mun, Akhilesh Pandey, Keira E. Mahoney, Jeffrey Shabanowitz, Satya Saxena, Benjamin C. Orsburn

**Affiliations:** 1 Optys Tech Corporation, Shrewsbury, MA 01545, USA; amol.prakash@optystech.com (A.P.); manuvarkey1987@gmail.com (M.V.); 2 McGill University Health Center, Montreal, QC H4A 3J1, Canada; proteomics.rimuhc@mcgill.ca; 3 Aptagen LLC, Philadelphia, PA 17407, USA; thedigitalchemist@protonmail.com; 4 Center for Proteomics and Metabolomics, Leiden University Medical Center, Albinusdreef 2, 2333 ZA Leiden, The Netherlands; y.mohammed@lumc.nl; 5 Department of Biochemistry and Molecular Genetics, Feinberg School of Medicine, Northwestern University, Chicago, IL 60208, USA; young.goo@northwestern.edu; 6 Thermo Fisher Scientific, Grimes, IA 50111, USA; scott.peterman@thermofisher.com; 7 Department of Neurology, Johns Hopkins University, Baltimore, MD 21205, USA; am@jhmi.edu (A.M.); yyuan@jhmi.edu (Y.Y.); 8 Los Alamos National Laboratory, Los Alamos, NM 87504, USA; tglaros@lanl.gov; 9 Infection and Immunity Program & Department of Biochemistry and Molecular Biology, Biomedicine Discovery Institute, Monash University, Melbourne, VIC 3004, Australia; joel.steele@monash.edu (J.R.S.); pouya.faridi@monash.edu (P.F.); 10 Department of Biosciences and Bioengineering, Indian Institute of Technology, Mumbai 400076, India; shaswati.parihari@gmail.com (S.P.); sanjeeva@iitb.ac.in (S.S.); 11 The University of Texas Southwestern Medical Center, Dallas, TX 75390, USA; Joseph.Otto@UTSouthwestern.edu; 12 Leroy T. Canoles Jr. Cancer Research Center, Eastern Virginia Medical School, Norfolk, VA 23501, USA; NyalwiJO@EVMS.EDU (J.O.N.); SemmesOJ@EVMS.EDU (O.J.S.); 13 Department of Molecular Genetics, The Hospital for Sick Children, University of Toronto, Toronto, ON M5S 1A8, Canada; m.moran@utoronto.ca; 14 Mayo Clinic, Rochester, MN 55902, USA; Madugundu.Anil@mayo.edu (A.M.); Mun.Dong-Gi@mayo.edu (D.G.M.); Pandey.Akhilesh@mayo.edu (A.P.); 15 Department of Chemistry, University of Virginia, Charlottesville, VA 22901, USA; kem5tk@virginia.edu (K.E.M.); js4c@virginia.edu (J.S.); 16 Deurion LLC, Ellicott City, MD 21041, USA; saxenasatya@gmail.com; 17 Department of Pharmacology and Molecular Sciences, Johns Hopkins University, Baltimore, MD 21205, USA

**Keywords:** cloud computing, proteomics, CPTAC, proteogenomics, post-translational modifications, tumor proteomics, cancer

## Abstract

**Simple Summary:**

We reanalyzed a publicly available breast cancer proteomics dataset consisting of 122 human tumor samples using a scalable cloud computing workflow. By doing so, we were able to search these files against millions of known human sequence variants and hundreds of common post-translational protein modifications, thereby demonstrating the power of cloud computing to address proteomic data in a true biological context. We identified thousands of relevant sequence variants and PTMs, indicating that the original studies may have only scratched the surface of the true value of the CPTAC studies completed to date. We present the results of this reanalysis in a searchable web interface for community analysis and validation.

**Abstract:**

The Clinical Proteomic Tumor Analysis Consortium (CPTAC) has provided some of the most in-depth analyses of the phenotypes of human tumors ever constructed. Today, the majority of proteomic data analysis is still performed using software housed on desktop computers which limits the number of sequence variants and post-translational modifications that can be considered. The original CPTAC studies limited the search for PTMs to only samples that were chemically enriched for those modified peptides. Similarly, the only sequence variants considered were those with strong evidence at the exon or transcript level. In this multi-institutional collaborative reanalysis, we utilized unbiased protein databases containing millions of human sequence variants in conjunction with hundreds of common post-translational modifications. Using these tools, we identified tens of thousands of high-confidence PTMs and sequence variants. We identified 4132 phosphorylated peptides in nonenriched samples, 93% of which were confirmed in the samples which were chemically enriched for phosphopeptides. In addition, our results also cover 90% of the high-confidence variants reported by the original proteogenomics study, without the need for sample specific next-generation sequencing. Finally, we report fivefold more somatic and germline variants that have an independent evidence at the peptide level, including mutations in ERRB2 and BCAS1. In this reanalysis of CPTAC proteomic data with cloud computing, we present an openly available and searchable web resource of the highest-coverage proteomic profiling of human tumors described to date.

## 1. Introduction

The Clinical Proteomic Tumor Analysis Consortium (CPTAC) was established to perform in-depth proteomic analysis of tumor tissues previously analyzed by the Cancer Genome Atlas Program (TCGA) as well as independent tumor samples. Today, CPTAC has completed in-depth analysis of hundreds of patient samples with thousands of highly fractionated liquid chromatography mass spectrometry (LCMS) experiments available for public access. With multiplexed labels, each experiment can possess its own internal quality controls allowing both superior inter-batch and intra-batch quantitative analyses. The methods for analyzing CPTAC data have evolved over time and have been rigorously described throughout their evolution. That LCMS-based proteomics can achieve interlaboratory reproducibility when sample handling and processing are rigorously controlled has been the demonstration of a powerful contribution of CPTAC to the proteomics community [1]. Furthermore, by directly analyzing samples from TCGA, CPTAC developed an ideal environment for the combination of proteomic and genomic data. The application of “proteogenomics” to these datasets has demonstrated how complementary these technologies can be when used in tandem. The systematic presence of somatic mutations in breast cancer was established in some of the earliest completed breast cancer genomes [2]. The ultimate effects of these mutations have been elusive until recently when a CPTAC proteogenomic study revealed interplay between these mutations and dysregulation in central phosphorylation signaling cascades [3]. A recent work has shown this is not limited to breast cancer as demonstrated by the proteomic prioritization of somatic copy number variations reported in a CPTAC proteogenomic analysis of colon cancer [4]. While these studies have demonstrated the power of proteomic and genomic technologies when utilized in tandem, they have inadvertently highlighted the weaknesses of these independent workflows when performed in isolation using traditional software [3,5]. 

Unlike genomic and transcriptomic data analysis, the majority of proteomic data analysis is performed using software designed for and executed on desktop personal computers [6], although large studies do utilize high-performance servers (but still the same software). Proteomic data continue scaling in both breadth and overall depth, with many of today’s instruments achieving true sequencing speeds approaching 100 Hz, nearly 10-fold more than the best technology in 2015 [7,8]. Today, shotgun proteomics can routinely achieve proteomic coverage at a comparable depth within similarly comparable timeframes as multiplexed transcriptomics [9,10,11]. While desktop PC architecture may have been suitable for thorough interrogation of LCMS data generated at 1 Hz, the only way to process today’s data is through a series of compromises to limit the overall search space [12]. We have recently described the use of a scalable cloud computing workflow using the modern search engine Bolt for the reanalysis of proteomic data [6,13]. While other solutions for proteomic analysis through cloud computing exist today, these largely require the skills of a dedicated bioinformatician to execute successfully [14]. Bolt utilizes a client-side graphical user interface similar to those of desktop search engines that handles all steps of data parsing, uploading, downloading and visualization, with no input necessary from the end user. With the scalable cloud backend of Bolt, we can analyze LCMS files from any instrument vendor with the use of vast libraries containing millions of human sequence variants. In addition, Bolt can be used to simultaneously search for hundreds of common human post-translational modifications (PTMs) by pulling more computational space from the cloud. These searches are either impractical or simply impossible to complete using desktop computers and/or traditional search engines and limit the biological relevance of proteomic data analysis [13]. In 2020, OptysTech was awarded an NCI contract through program 75N91020C00011 to evaluate the post-translational modifications present and currently unidentified in the existing CPTAC data. We applied Bolt to reanalysis of one recent CPTAC breast cancer study that was evaluated with thorough proteogenomic analyses [15]. We report 802,820 peptide IDs across the 425 RAW files, which is an increase of 53% over the default CRDC pipeline IDs, and provide a simple web interface for community evaluation of these IDs along with the annotated spectral evidence. We also report that this is the most comprehensive study ever performed on breast cancer tumor samples, identifying 96% and 87% of high-confidence peptides from previous unfractionated and fractionated studies despite completely different sample preparation and instrumentation. We observed that tens of thousands of post-translational modifications were readily observed in this dataset without the need for chemical enrichment. These results are further supported by the identification of the same phosphorylated peptides in the phospho-enriched samples from the original study. Furthermore, by considering a compiled database of approximately 3.6 million human genomic sequence variants along with PTMs, we demonstrate that proteomic data can be analyzed independently of genomics to identify sequence variants with high confidence. In this analysis, Bolt successfully identified the majority of peptide variants described in the original analysis while adding nearly five times more novel peptide variants. An independent manual analysis was utilized to provide further confidence to the identification of these novel peptide isoforms. The end results presented here by combining the value of CPTAC data with cloud computing matching the curated human sequence libraries amount to the single most comprehensive proteomic characterization of human tumors described to date. 

## 2. Materials and Methods

### 2.1. Protein Databases Utilized in this Study

Table 1 summarizes all the protein sequences utilized in this study.

### 2.2. Data Used in This Study

All the data were downloaded from NCI Data Commons, (https://pdc.cancer.gov/pdc/study, 15 September 2021) using the identifier PDC000120. Tumor samples were taken from 125 patient samples, and adjacent normal tissue samples from 18 patients were used as control material. Three tumors were rejected due to low-quality RNAseq data, and so the proteomic analysis was performed on 122 tumors. TMT-10plex labeling was performed on all the samples, with 131 channels used as a reference pool constructed from 40 tissue samples (normal and tumor). In total, there are 425 TMT-10plex-labeled RAW files [15].

### 2.3. Description of the Bolt Parameters Utilized in This Study

Search engine Bolt utilizes a proteoform variant database of approximately 3.6 million sequences and 450 mass modifications encompassing all currently known common human post-translational modifications and mass shifts of single amino acid substitutions resulting from missense mutations. Table 1 summarizes the various databases used to create the proteoform database utilized in this study (Bolt directly combined them into a single database and removed redundancy). Bolt was run on a cloud server with 128 GB RAM and 40 CPU cores. While we used the Microsoft Azure cloud platform, this could be deployed on any other cloud platform or even on an in-house server. All the spectra were searched with ± 20 ppm mass tolerance for both precursor and product ions to match the original study parameters. In addition, one terminal partially tryptic cleavage event was allowed, along with four missed cleavage sites. The total search time was ~20 days, approximately 1 h per file.

### 2.4. Custom Genomic Data analysis of Nine Patient Samples

Whole exome sequencing (WES) and RNAseq .bam files were downloaded using dbGAP-controlled access for all the nine tumor and normal samples belonging to the 02CPTAC TMT plex files. In this analysis, the pooled control channel was ignored. These datasets were already aligned to the hg38 reference, and the index file (.bai) was also available on dbGAP. For each peptide variant identified by Bolt, a custom script was used to identify the hg38 genomic coordinate of the single nucleotide variant (SNV) that resulted into the single amino acid variant (SAAV). For some peptides, this translated into multiple possibilities due to homology of protein sequences. When this occurred all coordinates were utilized. Approximately 5% of the SAAVs lacked the appropriate genomic coordinates for calculation and could not be used in this reanalysis. A custom script was used to search for evidence of the variant peptides in each of the WES and RNAseq .bam files using six-frame translation around ± 10 nucleotide residues from the identified genomic coordinate of the SNV. Some peptides were initially mapped at the exon/intron boundary. Only the part of the peptide piece that contains the SAAV and is contained fully in one exon was used for search evidence. This custom script reports the total count of the observations of the variant peptide observed across all the 18 WES and nine RNAseq .bam files.

### 2.5. CPTAC Data Pipeline Used to Compare the Proteogenomic Results

All the 122 tumor samples were analyzed using WES on both tumor and matched normal samples. The MuTect software was utilized on the WES data for detection of somatic SNVs by comparing the tumor WES to the matched normal WES data. These steps were then followed by (a) copy number characterization employing AllelicCapSeg and ABSOLUTE and (b) variant rescue, annotation and filtering including deTiN including the MAF Panel of Normals (PoN) filter for filtering false-positive germline variants and common artifacts from somatic mutation calls. The proteogenomic database tool QUILTS v3.0 was then used to incorporate the germline and somatic nonsynonymous SNVs into a protein sequence database for each patient [17]. The resulting databases were combined into a single file along with the RefSeq human protein database. This resulting file is referred to in this text as the patient-specific database. This database contained 179,768 sequences derived from genomic SNV evidence. In the first search, all the spectra were searched against just the RefSeq human database at 1% FDR, with the unmatched spectra searched against the patient-specific database using Spectrum Mill. All the spectra were searched with ± 20 ppm mass tolerance for precursor and product ions and were fully tryptic with four missed cleavage sites. Static modifications were carbamidomethylation of cysteine and TMT10 labeling of lysine. Allowed variable modifications for whole proteome datasets were acetylation of protein N-termini, oxidized methionine, TMT10 labeling of peptide N-termini, deamidation of asparagine, hydroxylation of proline in proteo-genomic (PG) motifs, pyroglutamic and pyro-carbamidomethylation. The final SNV IDs were filtered by 1% FDR, length > 7 and spectral count > 2 TMT10 plexes for low-confidence hits. In total, this led to identification of 3444 single amino acid variants (SAAVs). The TMT ratio was then calculated for each PG event by first removing all entries with < 50% precursor purity and then taking the median of all the entries for those SNVs that explain that PG event. The ratios for all the PG events for a patient were then standardized by subtracting the centering factor and dividing by the scaling factor of the protein-level TMT ratios for that patient derived from the results of the RefSeq-only search.

### 2.6. CRDC Data Pipeline Used to Compare PTM/Proteomics Results

The default CRDC/PDC pipeline processes all PDC data and makes the peptide/protein results available on the PDC portal. This pipeline uses the MS-GF+ search engine on the RefSeq database. Partially tryptic peptides and oxidized methionine were allowed in the search parameters. 

### 2.7. Construction of the Bolt CPTAC Web Portal

Web portal is hosted on AWS (http://www.optystech.com/bolt.html, accessed on 15 September 2021) and utilizes the Angular stack. Besides the various protein and peptide annotations, this portal displays the annotated MS/MS spectrum from the Bolt viewer and also integrates with a previously published MS/MS spectra annotation tool, IPSA [18].

## 3. Results and Discussion 

### 3.1. Peptide IDs with Bolt

**Figure 1:**
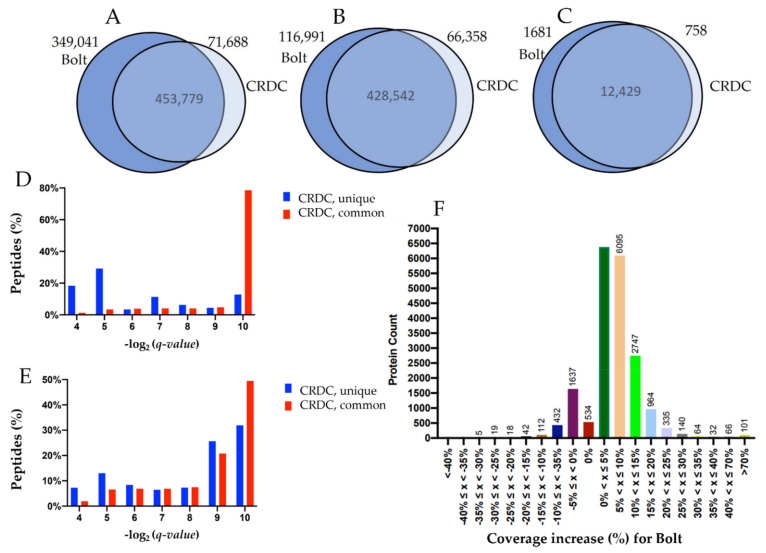
A comparison of Bolt peptide IDs to the original analysis. (**A**) Venn diagram comparing the total number of peptide IDs between the Bolt search results and the CRDC’s search results. (**B**) Venn diagram comparing the total number of unique peptide sequences between the Bolt search results and the CRDC’s search results. (**C**) Venn diagram comparing the total number of SwissProt protein IDs with at least two peptides between the Bolt search results and the CRDC’s search results. (**D**) Distribution of *q*-values of the CRDC’s unique vs. common peptide results. (**E**) Distribution of *q*-values of the Bolt’s unique vs. common peptide results. (**F**) Distribution of the protein sequence coverage increase by Bolt compared to CRDC.

Bolt identified a total of 802,820 peptide IDs across the 425 RAW files at 1% FDR, comprising 17.2 million spectra. They corresponded to 545,533 unique peptide sequences. In contrast, the CRDC pipeline identified 525,467 peptide IDs (494,900 unique peptide sequences) on these 425 RAW files. From Bolt’s unique results (453,779), approximately 85% were due to the expanded search space (large set of PTMs and variants). Figure 1A,B shows the Venn diagram comparing these results. Taking two peptide hits as a threshold to identify a protein, Bolt identified 14,110 proteins from SwissProt Canonical compared to 13,187 identified by CRDC (Figure 1C shows the Venn diagram comparing the results at the protein level, with at least two unique peptide IDs). As Bolt considers a vast search space, which is inclusive of the search space of CRDC’s search, we would expect it to find almost all high-confidence peptide IDs from CRDC. Figure 1D shows the distribution of the CRDC’s reported *q*-value for all its unique IDs as well as those that are common with Bolt. As expected, most of the unique IDs of CRDC are lower-scoring, with less than 10% of IDs having high confidence. Figure 1E plots the same distribution for the Bolt’s reported *q*-value for the unique IDs and the common IDs. In contrast with Figure 1C, almost 60% of the unique IDs from Bolt are high-confidence. Thus, not only did Bolt report 53% more peptide IDs; the majority of these unique IDs were also high-confidence. Overall, the CRDC pipeline annotated ~27% of the acquired MS/MS spectra, and Bolt annotated ~40% of all the MS/MS spectra (Supplementary Figure S1), which also shows that there is still a vast number of unannotated MS/MS spectra. Figure 1F shows the sequence coverage improvement (%) by Bolt for the SwissProt Canonical proteins compared to CRDC, where we report a coverage increase for 86% of the proteins compared to the decrease for 11% of the proteins. The instances where Bolt reported more than 70% coverage increase appear to be from the database differences (CRDC uses RefSeq, whereas Bolt uses both SwissProt and RefSeq). To the best of our knowledge, Bolt results are the largest collection of peptides reported from patient tumor samples for any cancer. The entire data are available for downloading as a Supplementary Materials File S1.

### 3.2. Evaluation of the Protein Sequencing Depth 

To get a sense of completeness of the data, we compared the Bolt’s peptide results at the peptide sequence level from this study with four other studies. Tyanova et al. studied 40 tumor samples with fractionation and a super SILAC sample made with cell lines, leading to 360 RAW files [19]. Tang et al. studied 65 breast tumors and 53 adjacent noncancerous tissues with extensive fractionation, leading to 118 RAW files [20]. Gomig et al. studied primary breast tumors, axillary metastatic lymph nodes and contralateral and adjacent breast tissues from seven patients, leading to 69 RAW files [21]. Lawrence et al. studied 20 breast cancer cell lines and four primary breast tumors, leading to 450 RAW files [22]. Table 2 presents the percentages of the Bolt’s overlap for each of these studies. In our comparisons, the Bolt result from this study identified 96% of the peptide IDs from the study that did not perform fractionation (PXD012431). When we compared the Bolt results to the studies that performed extensive fractionation, Bolt identified approximately 80% of the total peptide sequences, and 87% of the peptide sequences reported at 0.1% FDR (PXD005692). To put this in perspective, Bolt reported 86% overlap with the CRDC’s results on this study data, which we consider a remarkable result as these global studies used very different sample preparation techniques, instruments and data processing strategies. We conclude that the extensive fractionation procedures employed by CPTAC provide a comprehensive picture of the tumor environment. 

Another interesting result came from comparing the Bolt results to the studies that analyzed cell lines (PXD013455 and PXD009766), where we found approximately 70% overlap, highlighting the diverse expression profiles of cell lines and growing tumors noted by others [23,24,25].

### 3.3. Comparison with Proteogenomic Analysis

**Figure 2:**
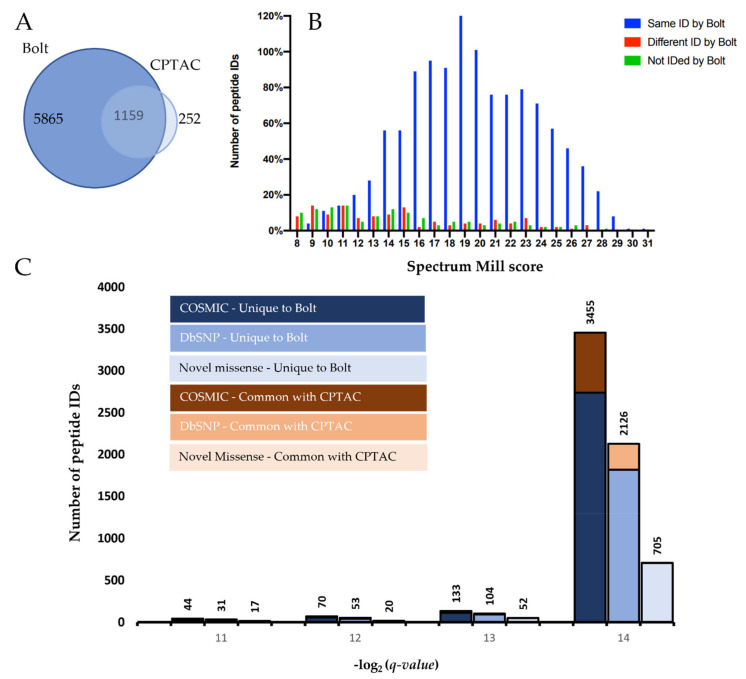
Comparing Bolt and CPTAC pipeline’s variant peptide IDs. (**A**) Venn diagram comparing the overall results between Bolt and CPTAC. (**B**) Spectrum Mill score distribution for the CPTAC’s peptide IDs that were also found by Bolt (blue), the peptide IDs that were matched to different spectra by Bolt (red) and the peptide IDs that were not reported by Bolt (green). (**C**) Distribution of *q*-values of the Bolt’s IDs that are unique as well as common with CPTAC, categorized into three categories: COSMIC, DbSNP and novel missense.

Next, for the proteogenomic comparison, we listed the Bolt’s peptide variant results and compared them to the CPTAC study. Bolt identified a total of 20,433 variant peptide IDs across the 425 RAW files by searching against the 7+ million database and all possible SAAVs. In comparison, the CPTAC study identified 3444 variant peptide IDs by searching against 179,768 somatic variant genomic sequences (patient-specific database). The CPTAC study then further filtered their results by filtering for those peptides that were observed in more than two TMT plexes (for low confidence) and with the length greater than 7. Using the same filters, Bolt reported 6991 variant peptides and the CPTAC study reported 1411 variant peptides. Figure 2A shows a Venn diagram comparing both of these sets of peptide IDs, whereby the Bolt’s list already contains 82% of the CPTAC IDs. If we consider only the CPTAC’s high-scoring IDs, then the comparison with Bolt improves to a total of 90% identification overlap. Figure 2B shows the CPTAC’s score distribution for all its peptide IDs categorized into three groups: peptide IDs that were also found by Bolt (blue), peptide IDs that were matched to different spectra by Bolt (red) and peptide IDs that were not reported by Bolt (green). We reported different spectrum match by Bolt as a separate category as many such matches appear to be due to the accurate assignment of the monoisotopic precursor mass (off by 1 Da). In these cases, the manual analysis shows a split with Bolt and Spectrum Mill, each making correct and incorrect identifications. Figure 2C demonstrates the *q*-value distribution of the Bolt’s IDs that are unique as well as common with the original analysis, broken into three categories: COSMIC, dbSNP and novel missense, showing that most of these are high-confidence.

The protein ERBB2 was highlighted by the authors as a protein of particular interest. Both the Bolt results and the CPTAC study identified the mutation P8T Isoform C. In addition, Bolt uniquely reported a peptide for the mutation P699L, which is a known COSMIC variant (COSM4476768). BCAS1 is another important protein for which we reported a similar observation. Both the Bolt results and the original analysis identified the mutation Q24K, but Bolt uniquely identified the additional mutations E356K (known COSMIC, COSM6403973), T270S (known population variant, rs182640580) and N76D (novel missense).

### 3.4. Evaluation of Discrepancies between the Bolt Results and the Original Analysis

**Figure 3:**
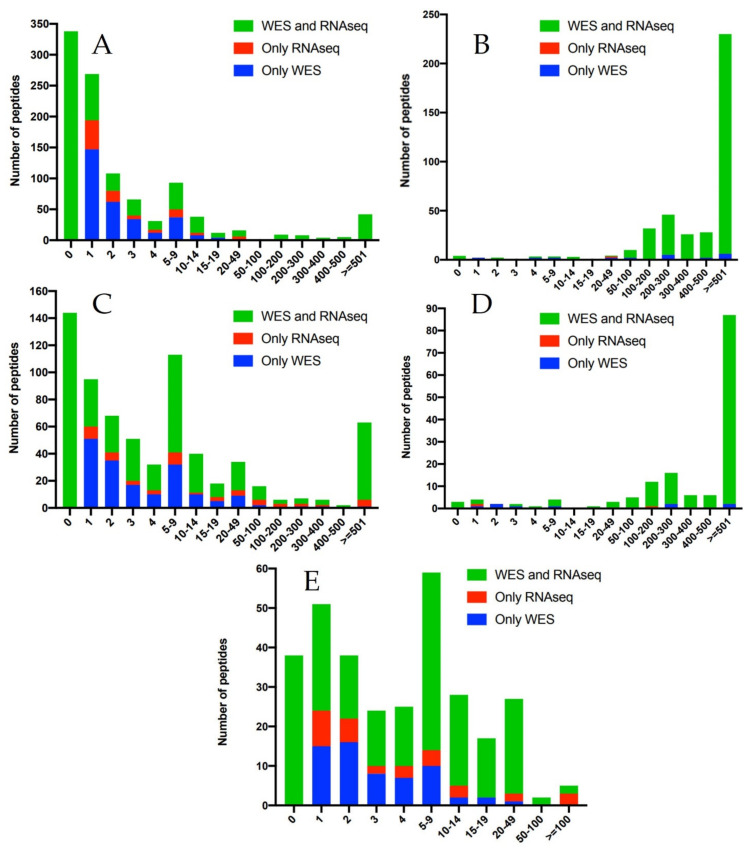
Distribution of the copy numbers observed with WES and RNAseq for the Bolt variant peptide IDs from the 02CPTAC TMT plex. The *x*-axis in all the plots is the genomic sequence copy number. (**A**) COSMIC variants that were not present in the CPTAC study; (**B**) COSMIC variants that were also identified in the CPTAC study; (**C**) dbSNP variants that were not present in the CPTAC study; (**D**) dbSNP variants that were also identified in the CPTAC study; and (**E**) novel missense variants that were not present in the CPTAC study. For all the plots, blue shows the copy number for the peptide IDs that were observed only in the WES data, red shows the copy number for the peptide IDs that were observed only in the RNAseq data and green shows the WES copy number for the peptide IDs that were observed with both WES and RNAseq.

We investigated these IDs in greater detail to determine why they were not identified using the CPTAC pipeline. Bolt’s 6991 peptide variants corresponded to 5422 unique variant events, which occur when multiple peptide sequences covering the same SAAV are clustered. We first filtered for those IDs that are present in the 02CPTAC TMT-plex which are all from 9 specific patient tumors samples. By doing so, we identified 3242 peptide IDs corresponding to 2595 variant events. We then searched for these variant peptide events in the raw genomic data from these 9 samples: 18 WES .bam files and nine RNAseq .bam files. The genomic copy number observed for these 2595 variant events in all of these 27 WES/RNAseq files is plotted in Figure 3. Figure 3A plots the distribution of the 1040 variant events that are from COSMIC and are not reported by the CPTAC study, and 3(b) plots the distribution of the 393 variant events that are from COSMIC and are also reported by the CPTAC study. This distribution clearly shows that most of the proteogenomic events reported by CPTAC had a high copy number and were also observed both with WES and RNAseq, whereas the IDs that are unique to Bolt have a generally lower copy number and were not universally observed with both WES and RNAseq. Figure 3C,D describes plots of the same data for dbSNP variants with 696 events unique to the Bolt search results, with 153 Bolt events that were in common with the original analysis. Although it generates a similar observation to the COSMIC plots, one marked difference is that Figure 3C has a large number of variants that were common to both WES and RNAseq and were not reported by CPTAC. This is an expected result as the CPTAC proteogenomic pipeline ignored population variants while generating the protein database. Figure 3E plots the distribution of the 314 novel missense variant events identified by Bolt, and this distribution has even lower copy numbers compared to COSMIC and dbSNP distributions.

### 3.5. Validation of Bolt IDs

To build confidence in the unique Bolt IDs, we performed an unbiased manual verification of peptide IDs with the assistance of researchers from the well-respected Don Hunt Laboratory at the University of Virginia [26]. In this analysis, we evaluated two peptides from each of the three categories, COSMIC, dbSNP and novel missense, that were identified in one RAW file. Bolt reported high-confidence *q*-values for each of these six peptides along with having a discriminatory ion that explains the site of mutation. None of these had been identified by the original analysis due to a low copy number from WES and RNAseq. Supplementary Table S1 lists these six peptides along with their proteomics and genomics annotation information, and Supplementary Figure S2 also shows the spectra for each of these six peptides. After manual sequencing, the Hunt Laboratory confirmed the IDs for each of these six peptides matching the Bolt results. As this was an independent and unbiased test, this helped provide additional confidence in the Bolt variant IDs in the absence of strong genomic evidence.

**Figure 4:**
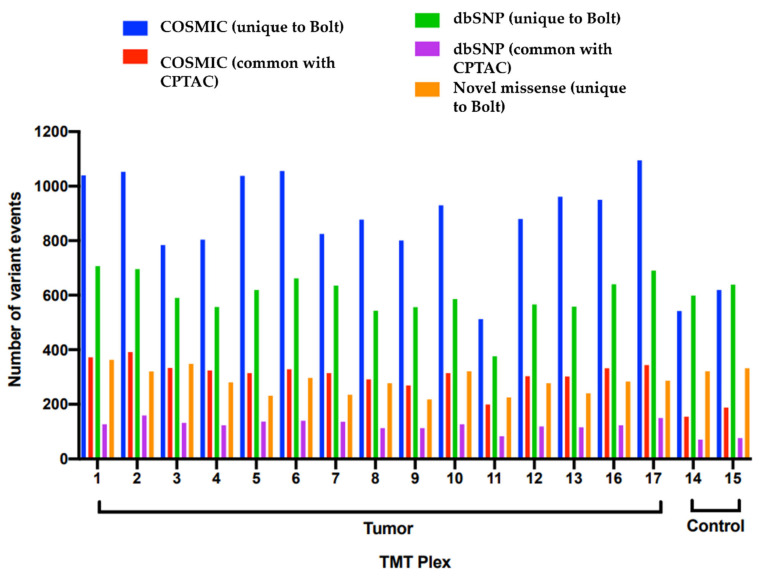
Mutation load of various TMT plexes for the different categories of variant events.

For quantitation, we first filtered all the variant IDs to those that we believed would yield robust quantitation. Variant events were only retained if they were observed in at least 10 TMT plexes with the further restriction that the corresponding protein had at least one canonical peptide observed. We employed two quantitation strategies. The first approach featured complete TMT-10plex quantitation for all these filtered variant events, where a variant event was considered significant if it had a TMT ratio greater than an arbitrary cutoff of 1.5 or less than 0.5. The normal sample TMT plexes were not used for any calculations in this strategy. The second strategy was spectral counting-based, where a variant event was considered significant if it was observed in at least 14 out of the total 15 tumor TMT plexes with a precursor ion with ≤ 50% isolation interference. Furthermore, the variant was required to be unobserved in either of the two control TMT sets, plex 14 and plex 15. The total number of variant events observed with these two strategies are listed in Supplementary Table S2. Given that both these quantitation strategies produced a relatively small list and also because the two strategies are so different in their approach, there was no overlap between the results of these two protein lists. The results show that there were many variant events reported uniquely by Bolt that present a stronger expression in the tumor samples compared to the control. Another methodology to evaluate these peptide variants is to look at their mutation load that they present in the various tumor vs. normal samples. Figure 4 plots the number of total variant events observed for each TMT plex categorized by tumor (plexes 1–13, 16 and 17) vs. normal (plexes 14 and 15). Both the set of COSMIC mutations uniquely identified by Bolt and the ones common with CPTAC provided clear discrimination between the tumor vs. normal sets. The other variants, both dbSNP and novel missense, appeared to be similar between the two sets.

**Figure 5:**
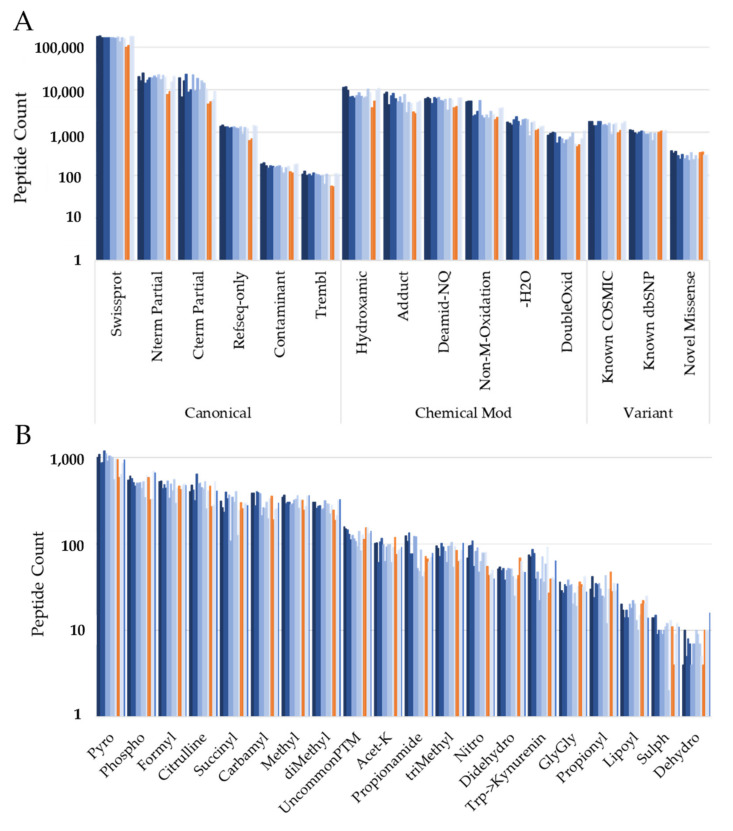
Distribution of various Bolt peptides for each TMT plex. Blue represents tumor TMT plex, and orange bars represent control TMT plexes in different categories: (**A**) canonical, chemical modification, sequence variant; (**B**) different biological modification categories.

Next, we investigated the modifications reported by Bolt. Figure 5A shows the various categories of the Bolt’s peptide IDs in the various TMT plexes. Blue bars represent the tumor TMT plexes and the two orange bars represent the control TMT plexes (14-plex and 15-plex). As expected, the canonical entries, fully tryptic and partially tryptic peptides account for the majority of the peptides, followed by peptides with various chemical modifications and sequence variants. One chemical modification was surprisingly common: hydroxylamine labeling of D, E and M amino acids, and this seems to be an artifact of the TMT quenching step. In fact, we observed this modification in 6% of the total peptides, whereas methionine oxidation was observed for only 5% of the total peptides. Thus, we need to be mindful of this modification when searching in TMT datasets. Figure 5B shows the various biological modifications observed for the different TMT plexes. In both Figure 5A and B, the peptide count is lower for control TMT plexes compared to tumor TMT plexes for almost all peptide classes. We believe this is an artifact of data handling as the original study also reported quantification issue with the control plexes. Next, we utilized the same quantitation strategies used for the variant study. We reported 22 modified peptides that were observed in at least 10 TMT plexes and had a normalized protein TMT ratio of 1.5 or more. This list contains five phosphorylated peptides. We also reported 73 modified peptides that were observed in at least 14 tumor TMT plexes and none of the two control TMT plexes.

Overall, Bolt identified 9149 phosphorylated peptides in the 122 tumor samples, and 7037 (77%) peptides out of those were also identified in the separate and independent CPTAC phospho-enriched tumor sample study. If we limit ourselves to those phosphorylated peptides that were observed in more than two TMT plexes, Bolt identified 4132 phosphorylated peptides, 3837 (93%) out of which were also identified in the phospho-enriched study. We believe this is a remarkable result as this suggests that these are high-confidence and abundant phosphorylated peptides that can be monitored even without additional sample enrichment. All the five of the previously mentioned phosphorylated peptides that Bolt reported as observed in at least 10 TMT plexes and having a TMT ratio of 1.5 or more were also reported in the enrichment study.

The list of modified and variant peptides identified in this study is available at http://www.optystech.com/bolt.html, accessed on 15 September 2021. To the best of our knowledge, this is the first such proteomic analysis portal which not only provides high-level annotations, but also the matching details such as MS/MS spectra annotations. This portal is also integrated with a previously published MS/MS annotation tool, IPSA [18]. Furthermore, it allows users to filter by different classes of modifications and peptide variants.

## 4. Conclusions

The future is bright for the proteomic technology. This may be most clearly demonstrated today with the recent proteomics screens leading to the development of the first inhibitors of mutant KRAS proteins [27,28,29]. With annual increases in proteomics sequencing depth and corresponding speed of analysis, clinical applications and personalized diagnostics are moving from theory to reality [30,31,32]. Today, hardware advances are not yet being matched with comparable advances in data processing. We hope that the results presented herein demonstrate a further proof of concept of the true power of proteomics when paired with modern and scalable cloud computing. 

We report sequence variants observed at the peptide level that are at the very low end of copy numbers from whole exon sequencing and RNA sequencing datasets. This is largely unsurprising today given the mounting evidence, including the results in the original CPTAC study re-evaluated here, that peptide abundance has little to no correlation with transcript abundance or copy number [33]. Current workflows typically rely on quality filters that are ultimately affected by the relative copy numbers of each transcript or genomic read [34]. We report herein the first evidence that proteomic data can be searched independently in a truly global manner to identify sequence variants that are transcribed and translated. We also observe that these variants have the expected mutation load discriminatory power between tumor and healthy samples. While we have manually validated a few of these, large-scale verification is not currently feasible for the millions of peptides identified in studies of this size. We do acknowledge that some of these could be false positives, but it is important to note that there are disagreements between WES and RNAseq as well [34,35]. If proteomics is considered with equal latitude, this will only result in the identification of new unique variants. If some of these variants prove to be biologically relevant and/or differentially expressed, then manual or secondary verification would be performed as a matter of course [36].

We also report identification of tens of thousands of post-translational modifications in these unenriched samples, which demonstrates the depth of biologically relevant data that can be mined from global proteomic data today. Modified peptides have the potential to be biomarkers of interest and the only limitation in their analysis consists in bioinformatic challenges.

We report five phosphorylated peptides that significantly altered expression levels in the tumor samples and were also observed in the phospho-enriched samples. In addition to biological modifications, we also report a technical artifact which was a result of the use of hydroxylamine reagent for TMT quenching. We report more hydroxylamine-modified peptides than those with methionine oxidation, demonstrating that any TMT study should consider hydroxylamine artifacts in their search parameters. The final takeaway of this large collaborative study must be that there is tremendous unexplored depth of potential biological findings hiding in plain sight in well-executed global proteomic studies such as those being performed by the researchers of the CPTAC initiative. 

## Figures and Tables

**Table 1:** A summary of the protein sequences utilized in this study.

Protein Database	Number of Protein Sequences	Version/Date/Source
Human SwissProt; Canonical + isoforms	42,414	UniProt, September, 2019
Human UniProt Trembl	53,211	UniProt, September, 2019
Common contaminants	269	cRAP database (gpm.org)
Known somatic variants (missense + nonsense)	2,537,773	February, 2020 (Lazar Lab) [16]
Known population variants (dbSNP)	1,042,598	dbSNP, July, 2020

**Table 2:** Bolt results compared with various other breast cancer studies whose data are publicly available. The percentages of peptide IDs common between each study and the data presented here are reported.

PRIDE Identifier	Type	FDR	Number of Fractions	Total Peptide IDs	Percentage Identified by Bolt
PXD009766	Tumor	1%	6	164935	77
	Tumor + SuperSILAC	1%	6	218107	70
PXD005692	Tumor	1%	17	101781	81
	Tumor	0.1%	17	70936	87
PXD012431	Tumor	1%	0	23483	96
PXD013455	Cell lines	1%	5	58305	69
	Tumor	1%	5	90336	78

## Data Availability

A web portal of all the peptide spectral matches described in this work is available for community interrogation at http://www.optystech.com/bolt.html, accessed on 15 September 2021.

## Supplementary Materials

The following are available online at https://www.mdpi.com/article/10.3390/cancers13205034/s1, Figure S1: Annotated vs. unannotated MS/MS spectra compared between (a) CRDC pipeline and (b) Bolt search result, Figure S2: Spectra of the selected 6 peptides, Table S1: Six peptides along with their proteomics and genomics annotation information, Table S2: Number of variant events that show significant fold-change by TMT ratio or by spectral count and observed in at least 10 TMT-plexes. File S1. Bolt results.
