# Supplementary material for: Reinspection of a Clinical Proteomics Tumor Analysis Consortium (CPTAC) Dataset with Cloud Computing Reveals Abundant Post-Translational Modifications and Protein Sequence Variants
Source: Cancers (Basel). 2021 Oct 9;13(20):5034. doi: 10.3390/cancers13205034 (PMC8534219; doi:10.3390/cancers13205034)
Supplement: Supplementary file 1 [file cancers-13-05034-s001.zip › cancers-1301780-suppl.pdf]

cancers

MDPI

Supplemental Materials

Reinspection of a Clinical Proteomics Tumor Analysis Consortium (CPTAC) Dataset with Cloud Computing Reveals Abundant Post-Translational Modifications and Protein Sequence Variants

Amol Prakash, Lorne Taylor, Manu Varkey, Nate Hoxie, Yassene Mohammed, Young Ah Goo, Scott Peterman, Abhay Moghekar, Yuting Yuan, Trevor Glaros, Joel R. Steele, Pouya Faridi, Shashwati Parihari, Sanjeeva Srivastava, Joseph J. Otto, Julius O. Nyalwidhe, O. John Semmes, Michael F. Moran, Anil Madugundu, Dong Gi Mun, Akhilesh Pandey, Keira E. Mahoney, Jeffrey Shabanowitz, Satya Saxena and Benjamin C. Orsburn

Table S1. Six peptides along with their proteomics and genomics annotation information

Peptide Variant	Charge	Scan Number	Observed MZ	Mass Error	Protein Name	Type	Canonical Sequence	Total copy number in all 9 samples	Copy number (best sample)
AVIDDFAAFVEK	3	44240	595.006	-0.46	P02768	COSM3605684	AVMDDFAAFVEK	44	13
DSYVGNEAQSK	2	10738	828.435	-0.46	P60709	COSM4602332	DSYVGDEAQSK	9	4
TDGETTDYAAPVK	2	16825	913.484	1.03	A0A0B4J1V0	Novel missense	TDGGTTDYAAPVK	45	19
TVMDDFAAFVEK	3	43757	610.996	0.95	P02768	Novel missense	AVMDDFAAFVEK	1	1
ASQGINSWLAWYQQK	3	39107	746.74	-1.02	P04430	dbSNP (rs372120393)	ASQGISNYLAWFQQK	0	0
EITALAPSTTK	2	21881	795.479	-0.22	P63267	dbSNP (rs199514403)	EITALAPSTMK	7	4

Table S2. Number of variant events that show significant fold-change by TMT ratio or by spectral count and observed in at least 10 TMT-plexes.

Count of variant events	COSMIC		dbSNP		Novel missense	
	Unique to Bolt	Common with CPTAC	Unique to Bolt	Common with CPTAC	Unique to Bolt	Common with CPTAC
Total	529	231	427	83	118	0
TMT ratio ≥ 1.5	5	1	12	2	6	0
TMT ratio ≤ 0.5	6	34	3	13	0	0
Observed in ≥ 14 tumor-TMT-plexes, but not in control-plexes	11	7	3	5	0	0

(a)

(b)

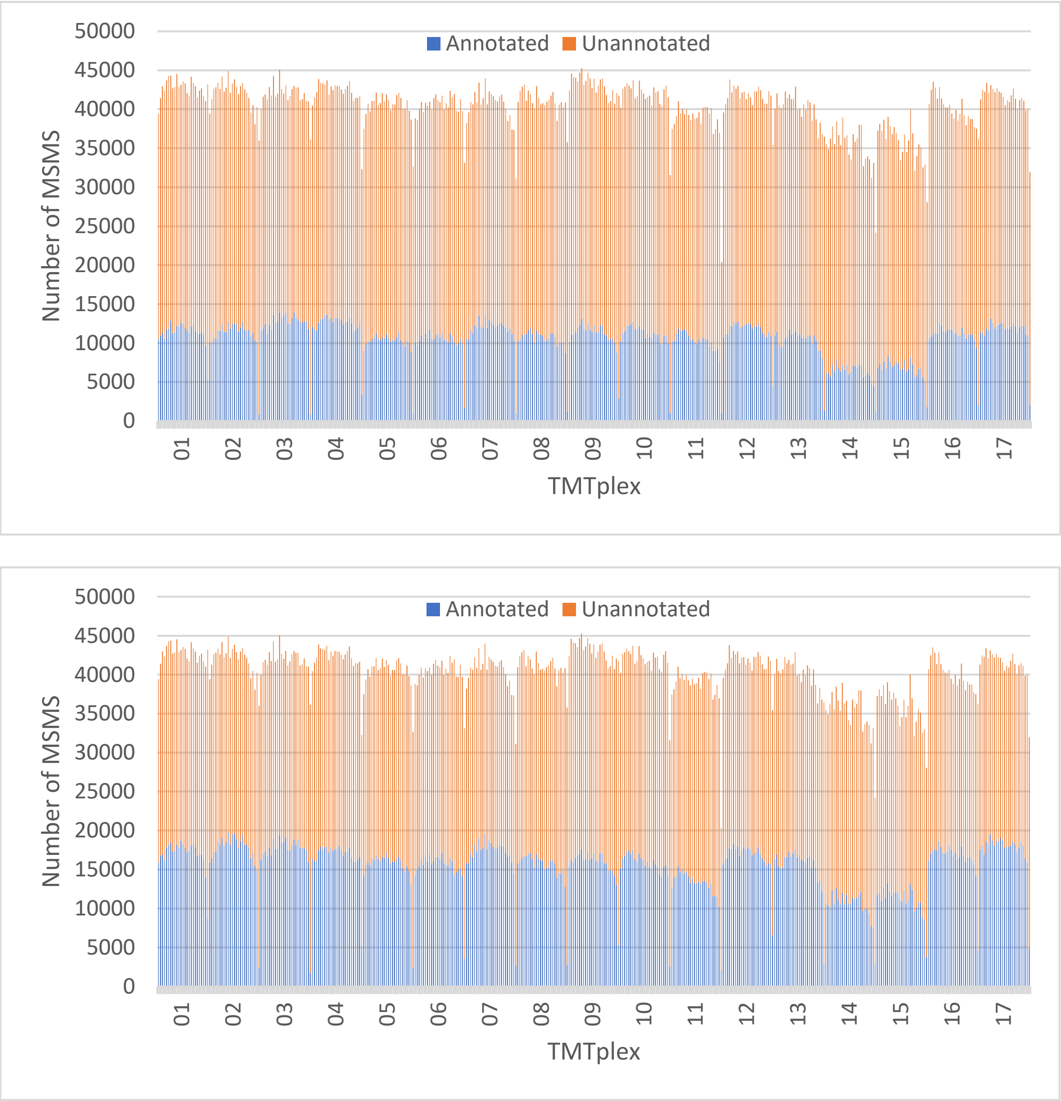

Figure S1. Annotated vs. unannotated MS/MS spectra compared between (**a**) CRDC pipeline and (**b**) Bolt search result.

(a) AVIDDFAAFVEK

(b) DSYVGNEAQSK

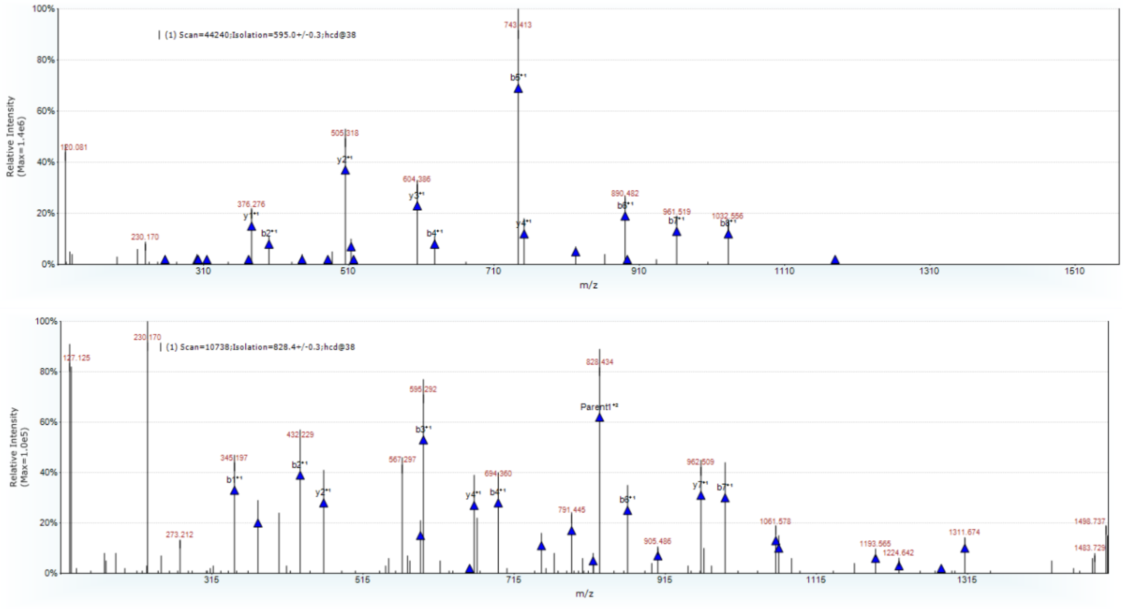

(c) TDGETTDYAAPVK

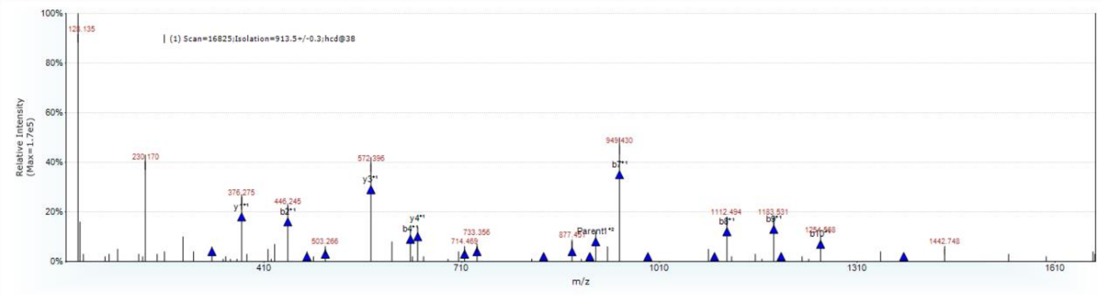

(d) TVMDDFAAFVEK

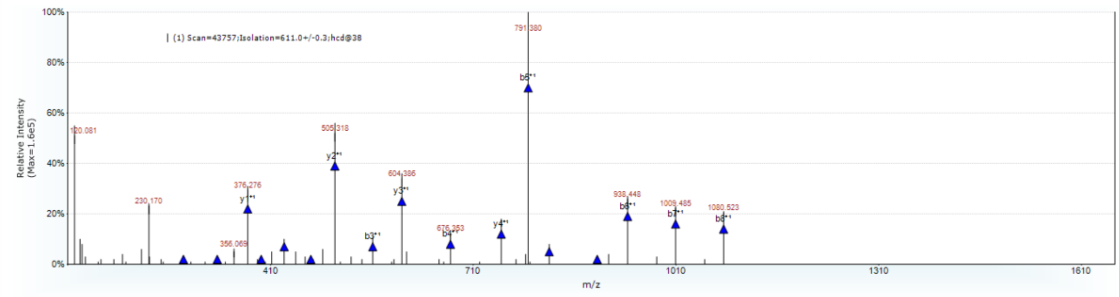

(e) ASQGINSWLAWYQQK

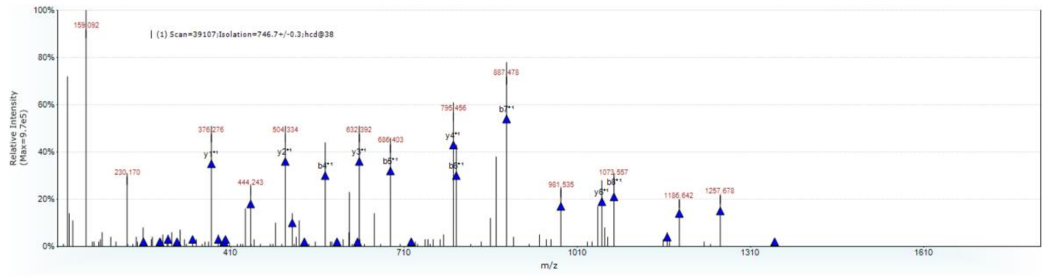

(f) EITALAPSTTK

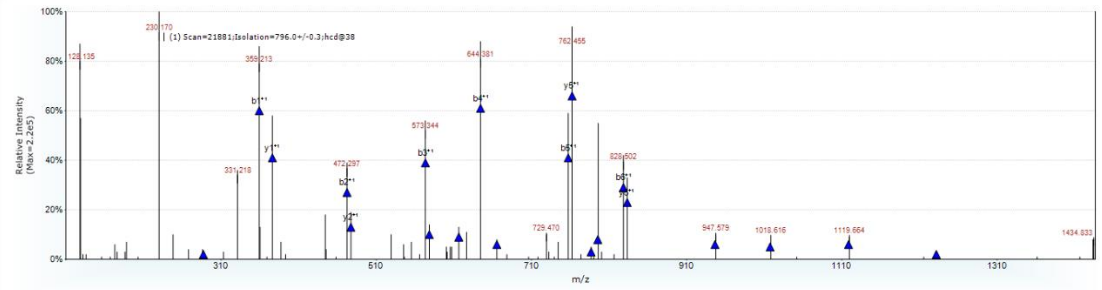

Figure S2. Spectra of the selected 6 peptides.
